# Factors leading to falls in transfemoral prosthesis users: a case series of prosthesis-side stumble recovery responses

**DOI:** 10.1186/s12984-024-01402-0

**Published:** 2024-07-13

**Authors:** Shane T. King, Maura E. Eveld, Karl E. Zelik, Michael Goldfarb

**Affiliations:** 1 Department of Mechanical Engineering, Vanderbilt University, Nashville, USA; 2 Department of Biomedical Engineering, Vanderbilt University, Nashville, USA; 3 Department of Physical Medicine and Rehabilitation, Vanderbilt University, Nashville, USA; 4 Department of Electrical Engineering, Vanderbilt University, Nashville, USA

**Keywords:** Trip, Gait biomechanics, Commercial prostheses, Amputation

## Abstract

**Background:**

Falls due to stumbling are prevalent for transfemoral prosthesis users and may lead to increased injury risk. This preliminary case series analyzes the transfemoral prosthesis user stumble recovery response to highlight key deficits in current commercially-available prostheses and proposes potential interventions to improve recovery outcomes.

**Methods:**

Six transfemoral prosthesis users were perturbed on their prosthetic limb at least three times while walking on a treadmill using obstacle perturbations in early, mid and late swing. Kinematic data were collected to characterize the response, while fall rate and key kinematic recovery metrics were used to assess the quality of recovery and highlight functional deficits in current commercially-available prostheses.

**Results:**

Across all participants, 13 (54%) of the 24 trials resulted in a fall (defined as > 50% body-weight support) with all but one participant (83%) falling at least once and two participants (33%) falling every time. In contrast, in a previous study of seven young, unimpaired, non-prosthesis users using the same experimental apparatus, no falls occurred across 190 trials. For the transfemoral prosthesis users, early swing had the highest rate of falling at 64%, followed by mid-swing at 57%, and then late swing at 33%. The trend in falls was mirrored by the kinematic recovery metrics (peak trunk angle, peak trunk angular velocity, forward reach of the perturbed limb, and knee angle at ground contact). In early swing all four metrics were deficient compared to non-prosthesis user controls. In mid swing, all but trunk angular velocity were deficient. In late swing only forward reach was deficient.

**Conclusion:**

Based on the stumble recovery responses, four potential deficiencies were identified in the response of the knee prostheses: (1) insufficient resistance to stance knee flexion upon ground contact; (2) insufficient swing extension after a perturbation; (3) difficulty initiating swing flexion following a perturbation; and (4) excessive impedance against swing flexion in early swing preventing the potential utilization of the elevating strategy. Each of these issues can potentially be addressed by mechanical or mechatronic changes to prosthetic design to improve quality of recovery and reduce the likelihood a fall.

**Supplementary Information:**

The online version contains supplementary material available at 10.1186/s12984-024-01402-0.

## Background

Stumbling (i.e., tripping) and falling is a frequent cause of injury among the adult population [1–4]. Studies have indicated that transfemoral prosthesis users are a high-risk group with a high prevalence of falls which can negatively impact both health and social engagement [5–10]. Short-term survey studies report that people with transfemoral amputation stumble between 2.5 and 4.4 times on average over a four-week period resulting in 1 to 1.8 falls [11, 12]. As such, individuals with transfemoral amputation experience a high fall risk.

Most current commercially-available knee prostheses are either passive or modulated-passive devices, which rely on the user’s hip motion to drive the ballistic motion of the prosthesis during swing phase [13]. In order to enable such ballistic motion, the prosthetic knee joint must be relatively low impedance. Low knee impedance, however, also exacerbates the effect of perturbations during a stumble [14–16]. Although the biological limb also exhibits similar low output impedance during swing, the biological limb can quickly and dramatically modulate its impedance via its musculature and neural reflexes, which mitigates the effect of stumble perturbations [17]. Some modulated passive devices, often called microprocessor knees (MPKs), feature stumble recovery mechanisms to reduce the likelihood of a fall during a stumble [11, 12, 14]. In the event of a perturbation during swing phase, the device can increase resistance to flexion, which reduces both the amount of knee deflection in the flexion direction during swing and the likelihood that the knee joint buckles upon loading as the user enters stance phase. Stumble recovery behavior likely plays an important role in assisting users following a perturbation as prior work has highlighted a fall disparity between MPK and non-MPK devices. A review paper found the C-Leg out performed other prostheses in certain safety metrics [18] while several multi-week survey-based studies have shown a decreased number of falls when using a C-Leg [11, 12] or an Ottobock 3E80 [19] over a non-MPK. A laboratory-based study also found individuals were less likely to fall following a variety of perturbations when using the C-Leg compared to non-MPKs (the Ottobock 3R80 and the Ossur Mauch Knee) [14]. While stumble recovery features can improve outcomes by restricting motion in flexion, such assistance is best suited for later in swing phase, and sometimes is still unable to prevent buckling due to the prosthesis’s configuration during loading (i.e., excessive knee flexion angle resulting in a knee flexion moment greater than can be effectively resisted) [15].

Non-prosthesis users generally rely on one of three reflexive recovery response strategies to help them recover following a stumble perturbation: the elevating strategy, lowering strategy, or delayed lowering strategy [20–22]. In the elevating strategy, which is often used in early swing, the foot is raised over the obstacle in the same swing phase as the perturbation by flexing the knee and hip. In the lowering strategy, which is often used in late swing, the foot is lowered prior to crossing the obstacle (i.e., swing is terminated prematurely). In the delayed lowering strategy, which is often used in mid swing, the foot is initially elevated but subsequently lowered. The three primary stumble recovery strategies have proven to be robust based on the low fall incidence in the adult, non-prosthesis user population. For example, in a previous laboratory study with seven non-prosthesis user participants, no falls occurred in the 190 trials conducted [20]. An underlying factor associated with each of the strategies employed by individuals with both biological limbs is active, high-impedance, coordinated control of the knee joint, both to quickly flex or extend while in swing phase and to support the weight of the person in any configuration in the subsequent stance phase. The elevating strategy was the most commonly observed strategy for non-prosthesis users in laboratory-based experiments when perturbations were applied in an even distribution across swing phase [20, 23]. However, there is not consistent evidence that the elevating strategy generally can be used in a reliable manner with passive knee prostheses, and as a result individuals with transfemoral amputation employ a number of compensatory strategies to recover from stumble instead [15, 24].

Various laboratory-based studies have examined the nature of stumble perturbation recovery responses in transfemoral prosthesis users. One study analyzed the response of four transfemoral prosthesis users (three C-Legs, one Ossur Mauch Knee) to a single obstacle perturbation in mid to late (50–66%) swing during overground walking and found all used the lowering strategy [15]. By comparison, non-prosthesis users have been found to use the elevating strategy up to 60–70% of swing phase [23]. Out of the four perturbations, one C-Leg user fell due to the knee buckling from excessive knee flexion upon loading the prosthesis (>50% body-weight support). Additionally, the non-MPK user was found to have greater knee flexion after the perturbation than the C-Leg users who recovered, potentially due to a lack of additional stumble recovery features. Another study characterized the response of eight transfemoral prosthesis users, including five C-Leg users and three non-MPK users (Ottobock 3R80, Ossur Total Knee, and Ossur Mauch Knee), to four prosthesis-side perturbations across swing phase (15–75%) using treadmill rope-blocking techniques [24]. This study found the distribution of recovery strategies used by the transfemoral prosthesis users across swing phase to generally replicate that of their non-prosthesis user controls, including attempted elevating strategies as late as 45–60% swing phase, which they concluded implied prostheses users would be able to take advantage of biomimetic stumble behaviors in a prosthesis. Additionally, this study reported no falls; however, use of handrails was allowed during the recovery, which could prevent potential falls and may have affected choice of recovery strategy. While the overground obstacle-based study did not note any use of the elevating strategy, both studies noted unique features in recovery behavior which varied from control participants. Hopping strategies, where participants jumped into a flight phase during the recovery response to clear the obstacle or adjust their base of support, and skipping strategies, where an extra step was taken by the perturbed limb prior to the contralateral limb ground contact, were both reported. The overground study only reported the hopping strategy during sound limb perturbations, while the treadmill study reported hopping during both prosthetic and sound limb perturbations and skipping during sound limb perturbations. The inconsistency in outcomes reported by the two studies may have resulted from the different perturbation techniques (i.e., obstacle versus rope-blocking), the amount of exposure to perturbations (i.e., one trial versus four), and/or availability of handrails. However, while the characterizations may vary, these studies were consistent in indicating that recovery responses across participants are varied and involve compensatory movement in the sound lower limb joints to assist in obstacle crossing following a perturbation.

Additionally, a recent survey report indicates that the preferential stumble recovery response of lower limb prosthesis users is to reach and grasp something to regain stability [25]. The preference for reach and grasp responses may be an additional indicator of a lack of confidence or difficulty in performing a successful stepping response such as those seen in non-prosthesis users. While a reach and grasp response may prevent falls in certain instances (i.e., when a grounded, graspable object is within reach), it is not an effective response in the absence of grounded apparatus (e.g., in the absence of a handrail).

The preliminary case series reported herein is intended to add to the limited body of knowledge describing stumble recovery in transfemoral prosthesis users. This study employs a stumble obstacle that must be cleared and was conducted while measuring full-body motion capture. These data are presented in juxtaposition with non-prosthesis user stumble recovery data to highlight key functional deficits in current prostheses which may leave transfemoral prosthesis users disadvantaged in their attempts to perform stumble recovery responses. In addition to the identification of the potential causes of recovery deficits, prosthetic design interventions are proposed that could potentially improve recovery outcomes. Prosthesis-side perturbations are the focus of this work, while the complementary sound side perturbation data is presented in [26].

## Methods

**Fig. 1:**
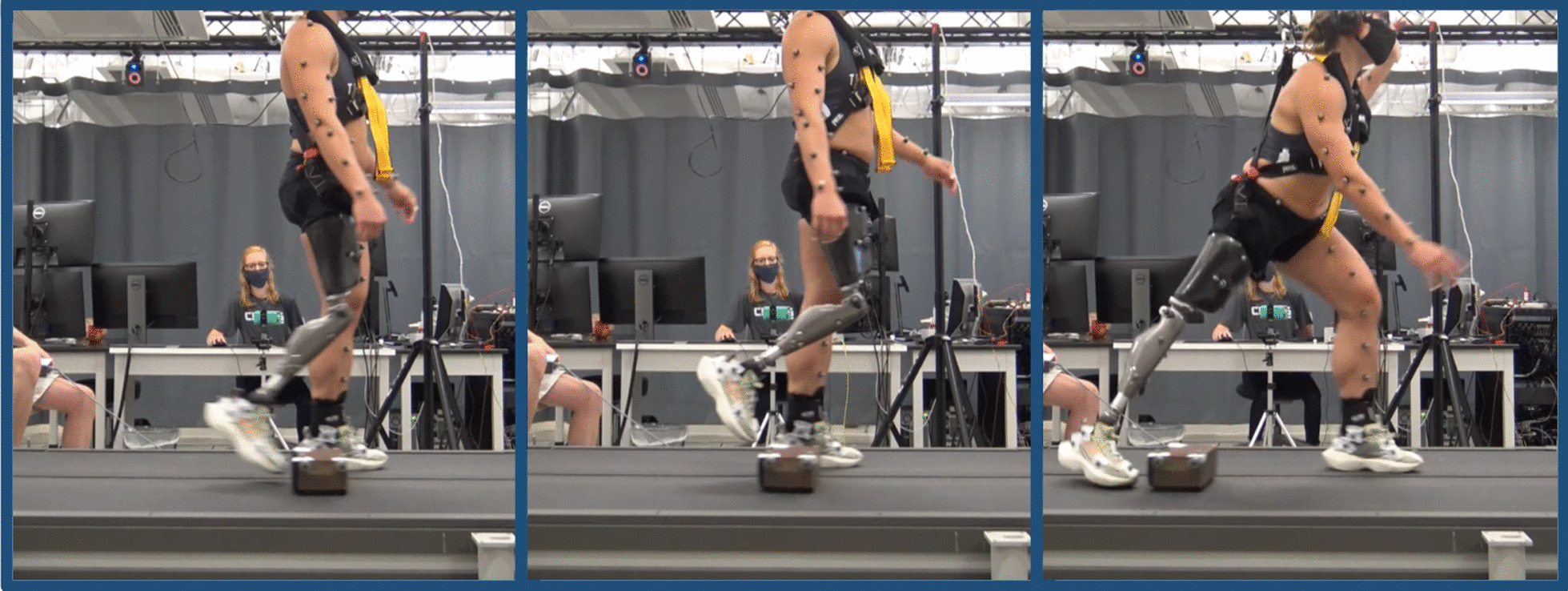
Experimental setup and a representative stumble perturbation recovery strategy. The participant uses the delayed lowering strategy in response to an early swing perturbation and successfully recovers. The first frame depicts the time of perturbation, the second frame depicts the initial elevating portion of the recovery strategy, and the third frame depicts the lowering portion of the recovery strategy

The preliminary study involved six transfemoral prosthesis users. Inclusion criteria included an age of 18–65, unilateral transfemoral prosthesis use, a K-Level of K3 or greater, and the ability to walk on a treadmill at 0.8 m/s. Exclusion criteria included daily-use of a powered knee prosthesis and any additional neurological or musculoskeletal impairment which affects gait or limits lower limb joint range of motion such as stroke or fused joints in the contralateral limb. Each participant walked using their prescribed prosthesis on a force-instrumented, split-belt treadmill (Bertec, Columbus, USA) at 0.8 m/s with the handrails removed, while stumble perturbation obstacles were introduced using a stumble perturbation apparatus described in detail in a previous publication [20]. The treadmill speed of 0.8 m/s was selected based on walking speed preference feedback from participants during pilot testing and in an effort to match prior treadmill-based studies with transfemoral prosthesis users [24]. Video frames from an experimental trial are shown in Fig. 1.

The obstacles were deployed using a ramp-based system at the front end of the treadmill (not shown in Fig. 1) which deposited the obstacles on the treadmill with minimal vertical or horizontal impulse at a time computed by a predictive targeting algorithm that targeted specific time points during swing phase. At least one perturbation was applied to the prosthetic limb in each of three general time periods of swing phase (i.e., early, mid and late) via the obstacle perturbation apparatus, and at least three additional perturbations were applied to the sound side to avoid anticipation by the participant of which leg would be perturbed. Sound side data is presented in [26].

The order of the perturbations was randomized for each participant and each perturbation was applied randomly between 25 and 45 strides from the onset of each trial. Each participant wore several pieces of sensory occlusion equipment including dribble goggles to block the inferior visual field, as well as earbuds playing white noise and passive, noise-canceling headphones to block the sound of the obstacle’s release. Participants also performed Serial Sevens (i.e., counting backwards by seven from an initial seed number) as a distraction to further reduce anticipatory behavior [27]. Participants wore a load cell-instrumented, full-body harness to both prevent the participant from contacting the treadmill during a fall and to record total body-weight assistance provided. Additionally, shoes with rigid toes were provided to those who desired them for additional toe protection. Not all participants wore the protective shoes due to personal preference for ease of walking. The efficacy of the apparatus with respect to timing the perturbation and delivering the perturbation without perception by the individual was verified and reported in [20]. Videos of the perturbations from each trial are included with the supplemental material as Additional file 1.

During all trials, participants wore a full-body motion capture marker set which included foot, shank, thigh, pelvis, trunk, upper arm, and lower arm segments. Motion capture data were collected using a 10-camera, infrared motion capture system (Vicon, Oxford, GBR) at 200 Hz. Ground reaction force (GRF) data were collected via the treadmill force plates at a sampling rate of 1000 Hz. GRF and motion capture data were filtered with a zero-phase, 3rd order, low-pass Butterworth filter with a cut-off frequency of 15 Hz and 6 Hz, respectively. Kinematics were computed using Visual 3D inverse dynamics software (C-Motion, Germantown, USA) which were then analyzed using MATLAB (MathWorks, Natick, USA). Data from young, unimpaired, non-prosthesis user controls were collected using the same methodology from a previous experimental data set of 190 trials across 7 participants reported in [20], all recorded at 1.1 m/s. The trials were used to provide an average range of response in the control group. However, it is important to note that the control trials were conducted at a faster treadmill speed relative to the trials reported herein (i.e., the control trials were conducted at 1.1 m/s, relative to the 0.8 m/s employed for transfemoral prosthesis user participants). The experimental protocol used in these studies was approved by the Vanderbilt Institutional Review Board, and all participants gave their informed written consent.

Swing percentage of the perturbation was determined by estimating the percentage from toe off to the timing of the perturbation, which was detected via a spike in the anterior-posterior GRF data during the onset of the perturbation. Since the overhead harness prevented falls (i.e., contact with the ground), a fall was instead defined as > 50% body-weight support in the harness at any point during the recovery [15, 28]. In cases when force plate crossover steps occurred following the perturbation, vertical foot center of mass (CoM) velocity was used instead to approximate heel strike and toe off [29]. The primary recovery strategy was determined by observing the trajectory of the swing foot CoM following the perturbation, with elevating defined by the foot moving upwards and crossing over the obstacle, lowering defined by the foot moving downwards, and delayed lowering defined by the foot moving upwards briefly then back downwards without crossing the obstacle [20]. Additionally, hopping and skipping sub-strategies were noted. Hopping was reported if the participant went into flight phase (i.e., both feet off the ground) during the initial recovery response (i.e., within the first two ground contacts) instead of alternating steps between limbs [15, 24]. Skipping was reported if the perturbed limb took an additional step prior to the contralateral limb ground contact without entering flight phase [24]. Unlike previous studies, these features are indicated as sub-strategies to better differentiate the initial responses since hopping and skipping may occur in addition to a primary recovery strategy [26].

Since the definition of a fall in this work is by necessity artificial and binary, a set of recovery outcome metrics were computed to provide a more continuous measure of the extent of perturbation and efficacy of recovery. These metrics are based on previously published indicators of quality of stumble recovery and/or predictors of falls [28, 30, 31]. These recovery metrics are broken into two groups. First, two trunk metrics were computed: (1) peak trunk angle during the recovery, and (2) peak trunk angular velocity during the recovery, both of which describe the ability of the participant to maintain their trunk control following the impulse of the perturbation and therefore control over the CoM. Second, two lower limb metrics were computed: (1) distance from the full-body CoM to perturbed foot CoM at ground contact after the recovery (referred to herein as forward reach) and (2) prosthetic knee angle at first ground contact after the perturbation. A complementary lower limb metric was also computed, (3) peak thigh abduction during the recovery, which is used to indicate the extent of compensatory action taken by participants (i.e., hip circumduction), rather than a direct assessment of quality or likelihood of recovery. Forward reach describes the ability of the participant to expand their base of support and recapture control of their CoM. Knee angle at ground contact demonstrates the prosthesis’s susceptibility to buckling in stance phase after the perturbation due to an increased flexion moment arm. Previous works have reported that greater than $30^{\circ }$ knee flexion at ground contact increases the likelihood of the knee buckling [15]. Thigh abduction is an indirect measure of hip circumduction to describe the amount of compensation being used to cross the obstacle due to the lack of direct knee control.

All metrics, except knee angle, were normalized to the values at the time of the perturbation. Trunk metrics were measured during the recovery response, which was limited to the time at which the second ground contact was made with the prosthetic limb following the perturbation. The second ground contact was used since this was where the bulk of the recovery occurred during a successful response using any of the three primary recovery strategies (i.e., elevating, lowering, or delayed lowering). By looking across the total window of the most critical portion of the recovery response, the peak magnitude of the effect of the perturbation on the trunk can be observed to demonstrate the quality of the response to the perturbation via CoM control. Forward reach was measured at ground contact after crossing the obstacle (i.e., first ground contact for the elevating response and second ground contact for the lowering responses) and was additionally normalized to participant height. Thigh abduction was measured during the stride that crossed the obstacle (i.e., first stride for the elevating response and second stride for the lowering responses).

Statistical analyses were used to indicate the extent to which recovery outcome metrics were significantly different between the transfemoral prosthesis user group and the control group. All statistical analysis is performed using the non-parametric Wilcoxon ranked-sum test with statistical significance indicated at p < 0.05.

## Results

Participant demographic data is presented in Table 1. Participants (5 males/1 female) were a mean age of 41 ± 13 with 19 ± 17 years of prosthesis use on average. All participants except Participant 5 (K3) self-reported a physician-prescribed K-Level of K4. Note that of the six participants, four used MPKs (all were the Ottobock C-Leg 4), and two used non-MPKs (one an Ottobock 3R80, a monocentric, rotary hydraulic knee, and one a Blatchford KX06, a four-bar, polycentric knee with a hydraulic cylinder). As such, any observations made regarding MPK behavior in this paper apply solely to the C-Leg.

**Table 1:** Participant demographic information

Participant	Age	Sex	Prosthesis side	Etiology	Years of prosthesis pse	K-level	Prescribed prosthesis
Participant 1	62	Male	Right	Trauma	49	K4	Ottobock C-Leg 4
Participant 2	42	Male	Left	Trauma	14	K4	Ottobock 3R80
Participant 3	28	Female	Right	Congenital	27	K4	Ottobock C-Leg 4
Participant 4	32	Male	Left	Trauma	4	K4	Blatchford KX06
Participant 5	50	Male	Left	Infection	5	K3	Ottobock C-Leg 4
Participant 6	30	Male	Left	Trauma	12	K4	Ottobock C-Leg 4

**Fig. 2:**
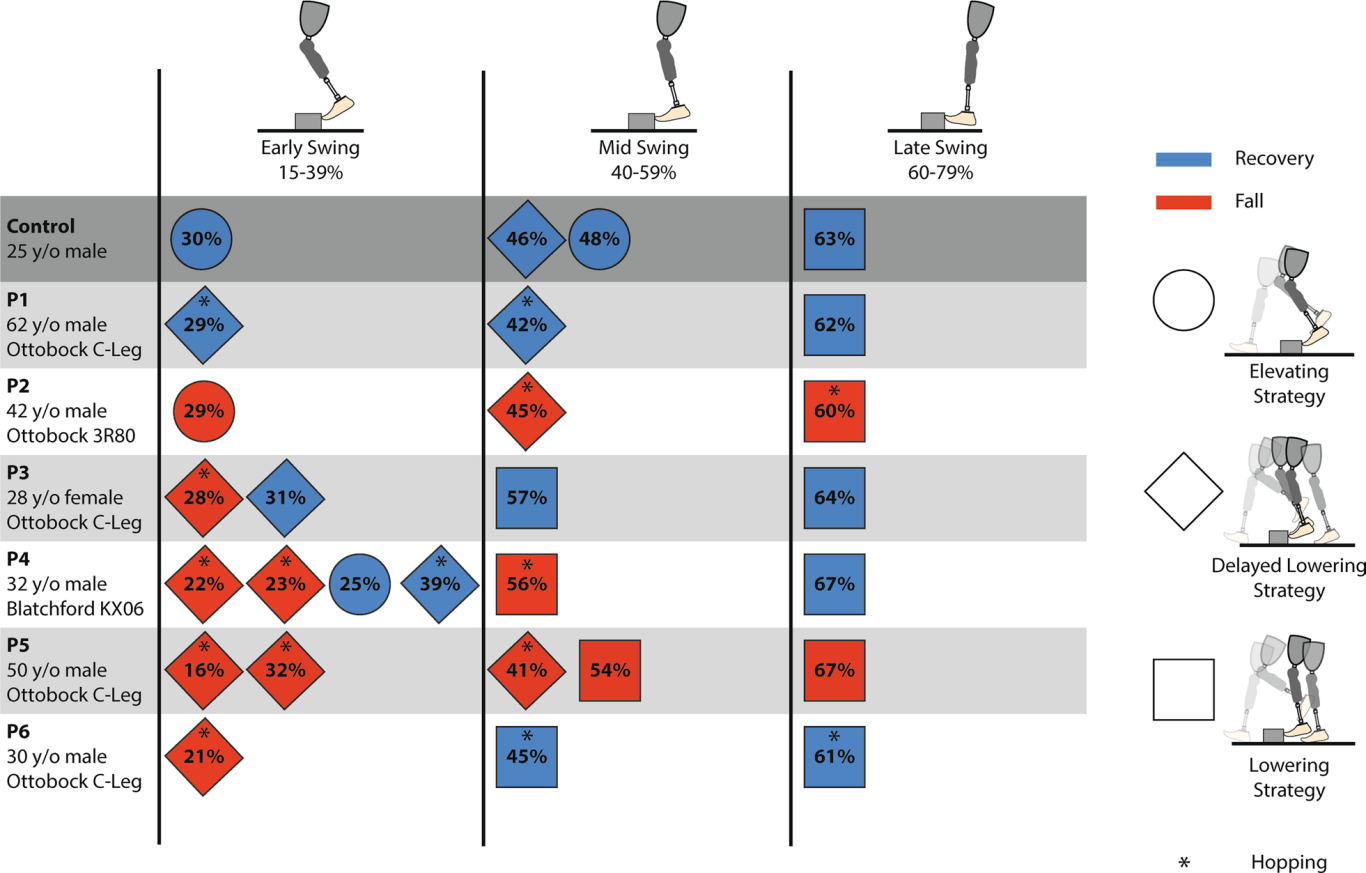
Graphical table of recovery outcomes (i.e., recovery versus fall), recovery strategy used, and perturbation swing percentage for every trial from each participant. The rows of the chart contain the information for each perturbation for a given participant with the top row depicting representative trials from a control participant. The columns contain early (15–39%), mid (40–59%), and late (60–79%) swing perturbations from left to right. Blue represents a successful recovery and red represents a fall. A circle represents the elevating strategy, a diamond represents the delayed lowering strategy, and a square represents the lowering strategy. An asterisk indicates the presence of hopping during the recovery response

The results of the trials are presented in terms of when the perturbation occurred in swing phase, specifically as either early, mid, or late swing, which correspond respectively to 15–39%, 40–59%, and 60–79% of swing phase. These categories were selected because, in the control group, early swing generally corresponds to the use of an elevating strategy; late corresponds to a lowering or delayed lowering strategy; and mid typically corresponds to the use of an elevating or delayed lowering strategy. Using the definition of a fall as previously stated, Fig. 2 indicates the distribution of falls versus recoveries (i.e., blue versus red) for each participant and each trial. The shape indicates the recovery strategy used with the swing percentage at which the perturbation occurred depicted inside of it. Recoveries which involved the hopping sub-strategy are indicated by an asterisk. No recoveries involved the use of the skipping sub-strategy. Across the six participants, 13 of the 24 (54%) trials resulted in a fall with all but one participant (83%) falling at least once and two participants (33%) falling every time. Representative control results are shown in the first row of Fig. 2, however, the seven controls never fell across 190 trials at a higher walking speed [20]. For the transfemoral prosthesis users, early swing had the highest rate of falling with 7 out of 11 (64%), followed by mid-swing with 4 out of 7 (57%), and then late swing with 2 out of 6 (33%).

### Early swing

Five out of six participants fell at least once during an early swing perturbation (83%). Seven of eleven total trials resulted in a fall, which is the highest rate of falls compared to mid and late swing perturbations at 64%.

#### Recovery strategies and kinematic data

**Fig. 3:**
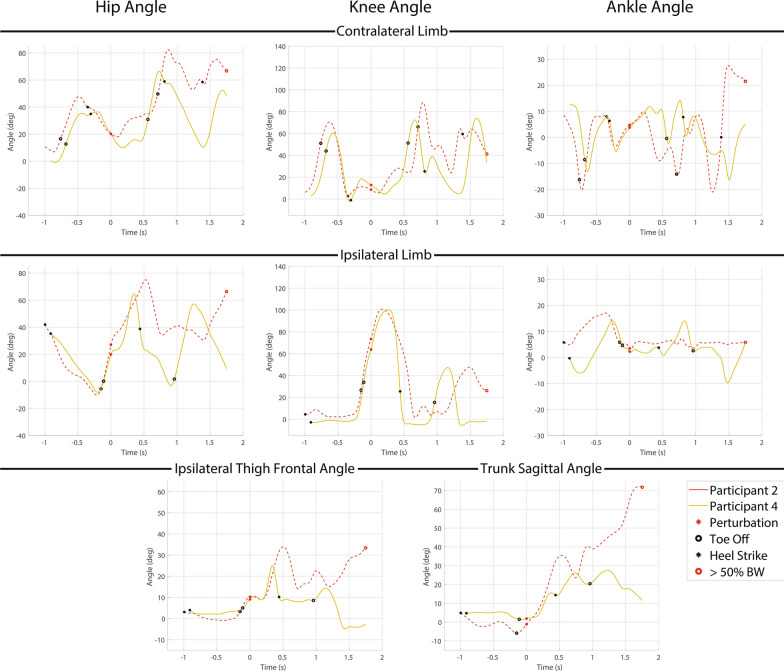
Kinematic data for early swing perturbation elevating strategy responses. Each plot depicts a different kinematic variable with the first row depicting the contralateral limb (i.e., support limb) hip, knee and ankle angle from left to right, the second row depicting ipsilateral limb (i.e., perturbed limb) hip, knee, and ankle angle from left to right, and the third row depicting ipsilateral thigh frontal plane angle (i.e., abduction) and trunk sagittal plane angle from left to right. Warm colored lines (red and yellow) depict non-MPK users and cool lines (cyan, purple, blue and green) depict C-Leg users (not seen here). Solid lines represent a successful recovery, while dashed lines represent a fall. The red dots represents when the perturbation occurs, the black dots represent heel strikes, the black rings represent toe offs, and the red rings represent when a fall occurs (for Participant 2 the fall occurred several steps later so the fall marker is placed at the end of the displayed trajectory)

**Fig. 4:**
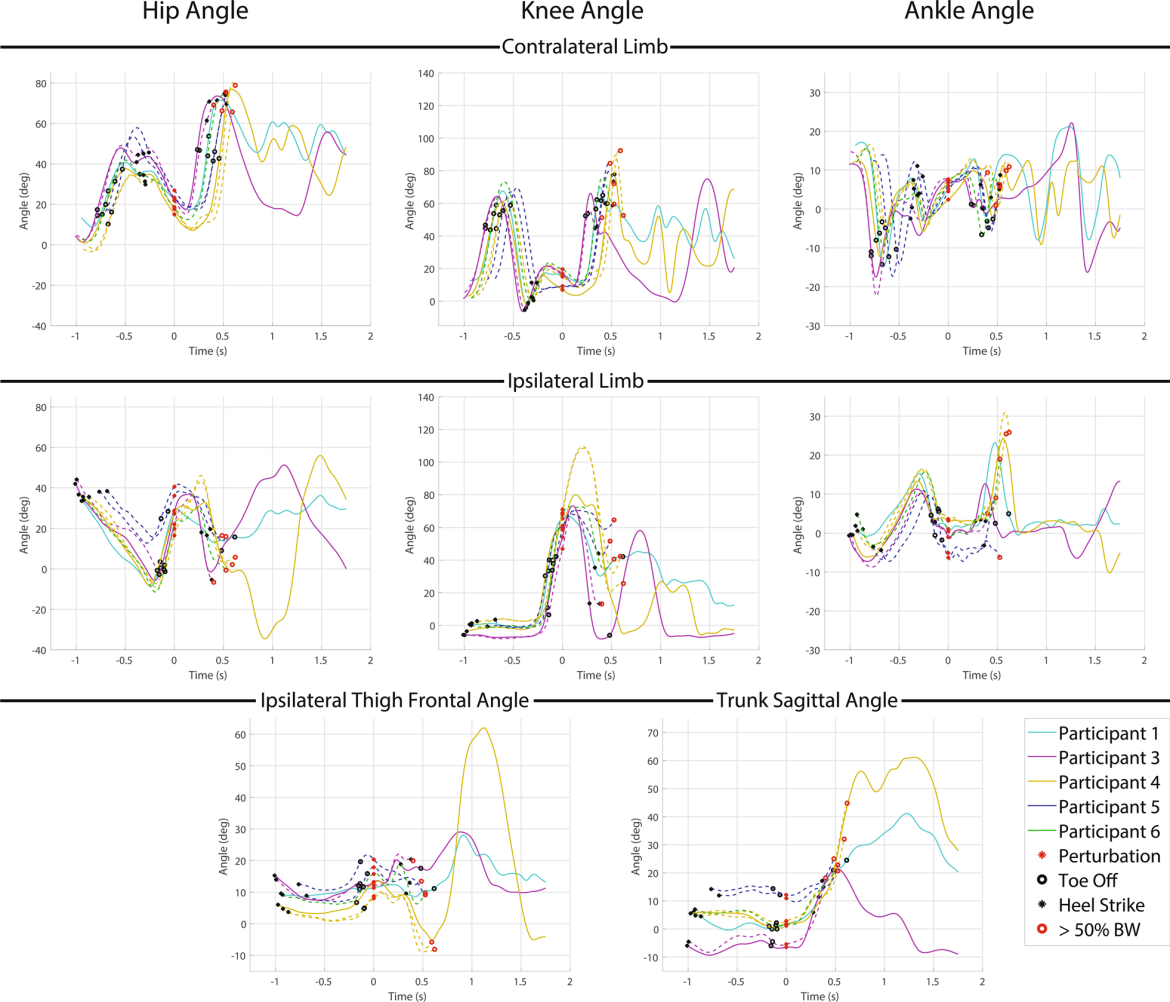
Kinematic data for early swing perturbation delayed lowering strategy responses. Each plot depicts a different kinematic variable with the first row depicting the contralateral limb (i.e., support limb) hip, knee and ankle angle from left to right, the second row depicting ipsilateral limb (i.e., perturbed limb) hip, knee, and ankle angle from left to right, and the third row depicting ipsilateral thigh frontal plane angle (i.e., abduction) and trunk sagittal plane angle from left to right. Warm colored lines (red and yellow) depict non-MPK users and cool lines (cyan, purple, blue and green) depict C-Leg users. Solid lines represent a successful recovery, while dashed lines represent a fall. The red circle represents when the perturbation occurs, the black circles represent heel strikes, the black rings represent toe offs, and the red rings represent when a fall occurs

Both elevating (2 times) and delayed lowering (9 times) strategies were used by the transfemoral prosthesis user participants. Hopping was observed in 8 of the 9 delayed lowering strategies, but neither of the elevating strategies. One of two elevating attempts resulted in a fall for one of the two participants who used it (50%) while 6 out of 9 delayed lowering strategies resulted in falls for four of the five participants who used it (67%). Hopping resulted in a fall in 6 out of 8 instances for four of the five participants who used it (75%). Kinematic trajectories for the elevating responses are presented in Fig. 3, while kinematic trajectories for the delayed lowering responses are presented in Fig. 4. Note the kinematic data shown is not normalized to the value at perturbation like the data in the following recovery metrics section.

The successful elevating strategy utilized high hip flexion ($64.7^{\circ }$) to cross the obstacle while the knee was flexed followed by hip extension to drive the prosthesis toward extension ($25.5^{\circ }$) prior to ground contact. For the unsuccessful elevating strategy, the participant fell several steps later due to the knee buckling after a poor landing in a slightly flexed position, which led to a loss of balance.

Successful recoveries across all strategies utilized thigh abduction (avg: $36.0^{\circ }$), indicating hip circumduction. However, the elevating strategy resulted in lower abduction ($24.9^{\circ }$) than the delayed lowering strategy ($62.0^{\circ }$) when successfully used by the same participant.

Non-fallers successfully increased knee extension before fully loading the prosthesis (avg: $24.8^{\circ }$) via quick hip extension during the elevating strategy or hip extension coupled with ground friction during the delayed lowering strategy. Additionally, most participants, successful or not, attempted to use sound side ankle plantarflexion to increase obstacle clearance.

One participant (P1) who successfully recovered using the delayed lowering strategy made ground contact with higher knee flexion ($35.4^{\circ }$), but avoided knee buckling by rotating his body and prosthesis in the transverse plane orthogonal to the motion of the treadmill to keep the knee joint out of the plane of motion while continuing to sidestep for several strides.

#### Recovery metrics

**Fig. 5:**
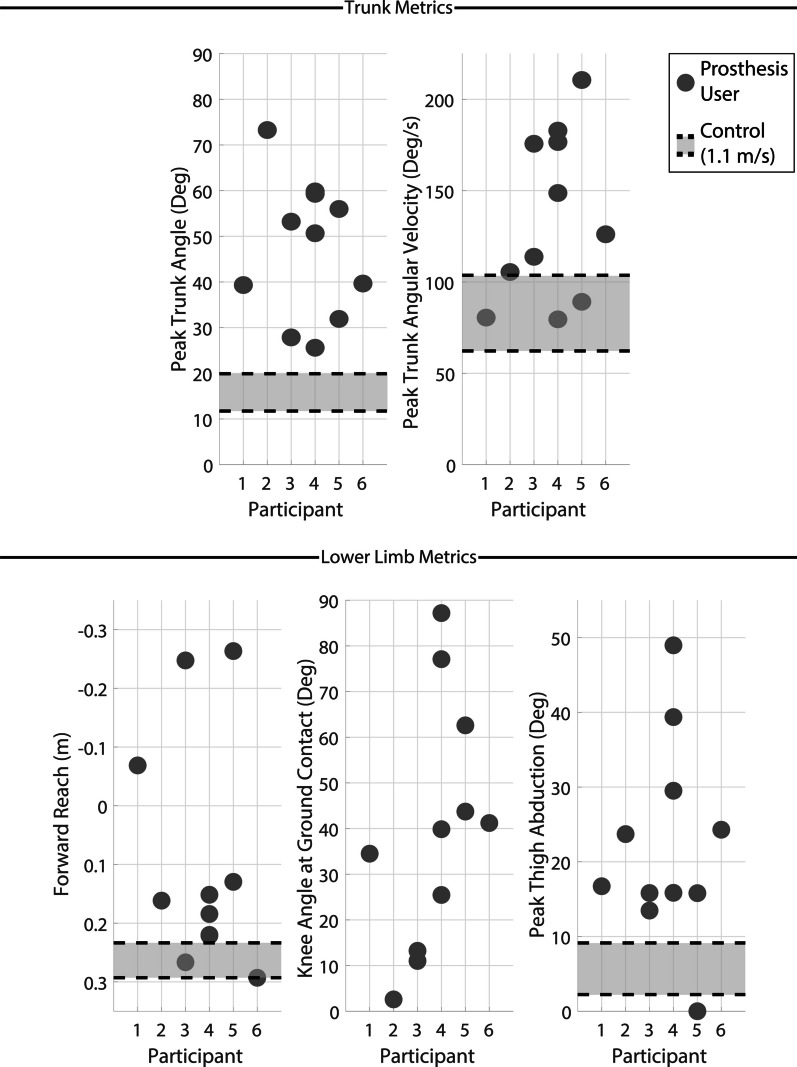
Key kinematic recovery outcome metrics for early swing perturbations. The top row displays peak trunk angle (left) and peak trunk angular velocity (right). The bottom row displays forward reach (body CoM to foot CoM, left), knee angle at first ground contact (middle) and peak thigh abduction (right). All metrics except knee angle are normalized to the value at the perturbation and are measured between the perturbation and the final recovery ground contact (first for elevating and second for delayed lowering and lowering). Knee angle is measured at first ground contact. Forward reach has an inverted vertical axis to maintain the visual trend across the plots (i.e., markers positioned lower on the plots indicate a more favorable outcome). The light grey region bordered in black dashed lines indicate the interquartile range of the control group data at 1.1 m/s from a previous study [20, 23]

Trunk recovery metrics are shown in the top row of Fig. 5 with control data for comparison. Lower values indicate greater trunk control and therefore higher quality of recovery and reduced likelihood of a fall. Transfemoral prosthesis users exhibited high trunk angle and angular velocity deviations following the early swing perturbations than from mid and late swing perturbations.

Lower limb recovery metrics are shown in the bottom row of Fig. 5 with control data for comparison. Spatially lower values indicate improved recovery responses or reduced compensation. Note for forward reach the y-axis has been inverted to maintain a consistent spatial trend as numerically higher values indicate improvement in this metric. Transfemoral prosthesis users demonstrated poor forward reach with many trials near or below 0 m (i.e., less forward reach corresponds to a lower ability to arrest forward momentum and adequately adjust the base of support). The knee angle at ground contact was variable, but most trials resulted in angles above $30^{\circ }$ with three trials greater than $60^{\circ }$, indicating increased risk of buckling as the prosthesis is loaded entering stance phase. Comparative control data is not included for knee angle as knee extension is easily controllable for individuals with a biological knee joint and not relevant to recovery. High peak thigh abduction was observed in most trials, suggesting transfemoral prosthesis users rely on abduction to attempt to extricate their foot from the obstacle following a perturbation. Both trunk and lower limb metrics for early swing are summarized in Table 2. Recovery strategies are abbreviated in all results tables as E, DL, and L for elevating, delayed lowering and lowering, respectively.

**Table 2:** Early swing perturbation outcomes

Participant	Fall?	% Swing	Strategy	Trunk ang	Trunk ang vel	Forward reach	Knee ang	Thigh abd
P1	No	29%	DL w/ hop	$39.3^{\circ }$	$80.5^{\circ }$/s	-0.07 m	$34.5^{\circ }$	$16.7^{\circ }$
P2	Yes	29%	E	$73.3^{\circ }$	$105.4^{\circ }$/s	0.16 m	$2.6^{\circ }$	$23.7^{\circ }$
P3	Yes	28%	DL w/ hop	$53.2^{\circ }$	$175.7^{\circ }$/s	-0.25 m	$13.2^{\circ }$	$13.5^{\circ }$
	No	31%	DL	$27.9^{\circ }$	$113.8^{\circ }$/s	0.27 m	$11.0^{\circ }$	$15.8^{\circ }$
P4	Yes	22%	DL w/ hop	$50.7^{\circ }$	$176.6^{\circ }$/s	0.22 m	$77.1^{\circ }$	$29.5^{\circ }$
	Yes	23%	DL w/ hop	$59.8^{\circ }$	$148.7^{\circ }$/s	0.18 m	$87.2^{\circ }$	$39.4^{\circ }$
	No	25%	E	$25.6^{\circ }$	$79.5^{\circ }$/s	0.15 m	$25.5^{\circ }$	$15.8^{\circ }$
	No	39%	DL w/ hop	$59.3^{\circ }$	$182.9^{\circ }$/s	0.22 m	$39.9^{\circ }$	$49.0^{\circ }$
P5	Yes	16%	DL w/ hop	$56.0^{\circ }$	$210.6^{\circ }$/s	-0.26 m	$62.6^{\circ }$	$0.0^{\circ }$
	Yes	32%	DL w/ hop	$31.9^{\circ }$	$89.1^{\circ }$/s	0.13 m	$43.7^{\circ }$	$15.8^{\circ }$
P6	Yes	21%	DL w/ hop	$39.7^{\circ }$	$126.1^{\circ }$/s	0.29 m	$41.2^{\circ }$	$24.3^{\circ }$

### Mid swing

Three out of six participants fell at least once during a mid-swing perturbation (50%). Four out of seven perturbations ended in a fall, giving mid swing the second highest fall rate after early swing with 57%.

#### Recovery strategies and kinematic data

**Fig. 6:**
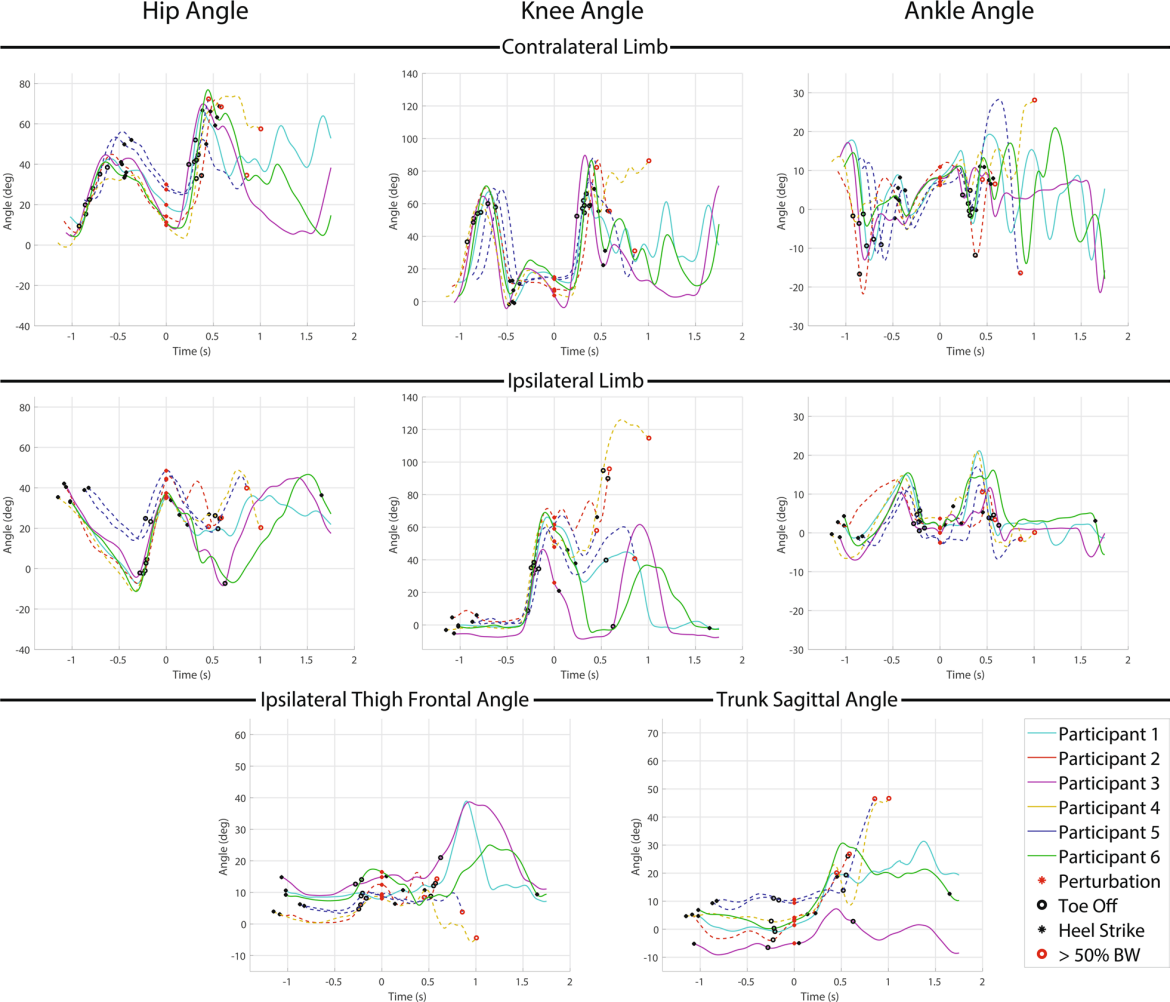
Kinematic data for mid swing perturbation delayed lowering and lowering strategy responses. Each plot depicts a different kinematic variable with the first row depicting the contralateral limb (i.e., support limb) hip, knee and ankle angle from left to right, the second row depicting ipsilateral limb (i.e., perturbed limb) hip, knee, and ankle angle from left to right, and the third row depicting ipsilateral thigh frontal plane angle (i.e., abduction) and trunk sagittal plane angle from left to right. Warm colored lines (red and yellow) depict non-MPK users and cool lines (cyan, purple, blue and green) depict C-Leg users. Solid lines represent a successful recovery, while dashed lines represent a fall. The red circle represents when the perturbation occurs, the black circles represent heel strikes, the black rings represent toe offs, and the red rings represent when a fall occurs

All recovery strategies were either delayed lowering (3 of 7) or lowering (4 of 7) strategies. Hopping was observed in all 3 delayed lowering strategies and 2 of the 4 lowering strategies. Three participants used the delayed lowering strategy and 2 resulted in a fall (66%), while four participants used the lowering strategy and 2 resulted in a fall (50%). Hopping resulted in a fall in 3 out of 5 instances (60%). Kinematic trajectories for the delayed lowering and lowering strategies are presented in Fig. 6. Successful recoveries were entirely from C-Leg users (2 lowering, 1 delayed lowering), all of whom utilized thigh abduction in the subsequent recovery step (avg: $34.2^{\circ }$) to circumvent the obstacle for a successful recovery. As with early swing delayed lowering strategies, hip extension was employed to extend the knee (avg: $29.4^{\circ }$) to prevent buckling in non-fallers compared to fallers. One participant (P1) again rotated sideways to avoided knee buckling as in the early swing phase perturbation.

#### Recovery metrics

**Fig. 7:**
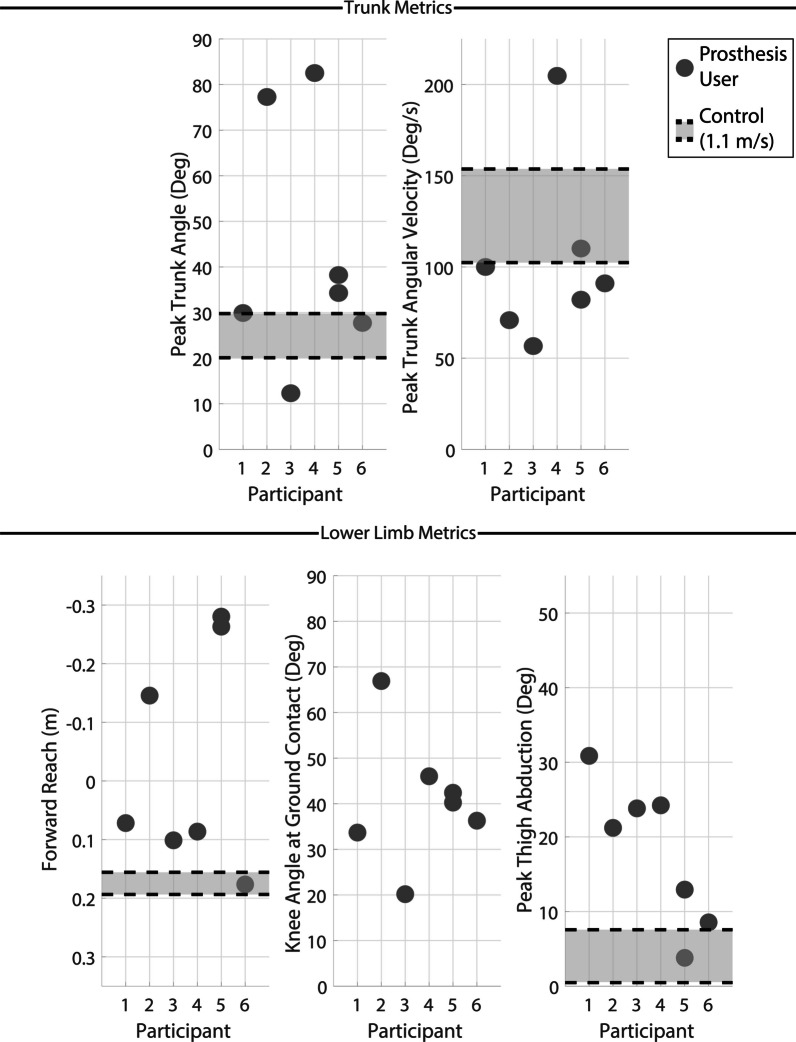
Key kinematic recovery outcome metrics for mid swing perturbations. The top row displays peak trunk angle (left) and peak trunk angular velocity (right). The bottom row displays forward reach (body CoM to foot CoM, left), knee angle at first ground contact (middle) and peak thigh abduction (right). All metrics except knee angle are normalized to the value at the perturbation and are measured between the perturbation and the final recovery ground contact (first for elevating and second for delayed lowering and lowering). Knee angle is measured at first ground contact. Forward reach has an inverted vertical axis to maintain the visual trend across the plots (i.e., markers positioned lower on the plots indicate a more favorable outcome). The light grey region bordered in black dashed lines indicate the interquartile range of the control group data at 1.1 m/s from a previous study [20, 23]

Trunk recovery metrics are shown in the top row of Fig. 7 with control data for comparison. Deviations in trunk metrics were less severe following mid swing perturbations compared to early swing, however, some participants still displayed high trunk angle deviation.

Lower limb metrics are reported in the bottom row of Fig. 7 with control data for comparison. Once again, a low forward reach is frequently observed for transfemoral prosthesis users, even greater than what was seen in early swing. Knee flexion at ground contact is high for most trials with all but one above $30^{\circ }$ and at high risk of buckling. High peak thigh abduction is also observed, indicating the necessity for circumduction to move around the obstacle during recovery. Both trunk and lower limb metrics for mid swing are summarized in Table 3.

**Table 3:** Mid swing perturbation outcomes

Participant	Fall?	% Swing	Strategy	Trunk ang	Trunk ang vel	Forward reach	Knee ang	Thigh abd
P1	No	42%	DL w/ hop	$29.9^{\circ }$	$100.0^{\circ }$/s	0.07 m	$33.7^{\circ }$	$30.9^{\circ }$
P2	Yes	45%	DL w/ hop	$77.3^{\circ }$	$70.8^{\circ }$/s	-0.15 m	$66.9^{\circ }$	$21.1^{\circ }$
P3	No	57%	DL	$12.3^{\circ }$	$56.6^{\circ }$/s	0.10 m	$20.2^{\circ }$	$23.8^{\circ }$
P4	Yes	56%	DL w/ hop	$82.5^{\circ }$	$204.8^{\circ }$/s	0.09 m	$46.0^{\circ }$	$24.2^{\circ }$
P5	Yes	41%	DL w/ hop	$34.3^{\circ }$	$82.0^{\circ }$/s	-0.28 m	$42.4^{\circ }$	$3.8^{\circ }$
	Yes	54%	L	$38.2^{\circ }$	$110.1^{\circ }$/s	-0.26 m	$40.2^{\circ }$	$12.9^{\circ }$
P6	No	45%	L w/ hop	$27.7^{\circ }$	$91.0^{\circ }$/s	0.18 m	$36.3^{\circ }$	$8.5^{\circ }$

### Late swing

Two out of six participants fell during late swing perturbations (33%). Two out of six perturbations resulted in a fall (33%), giving late swing the lowest fall rate of the three regions of swing phase analyzed in this study.

#### Recovery strategies and kinematic data

**Fig. 8:**
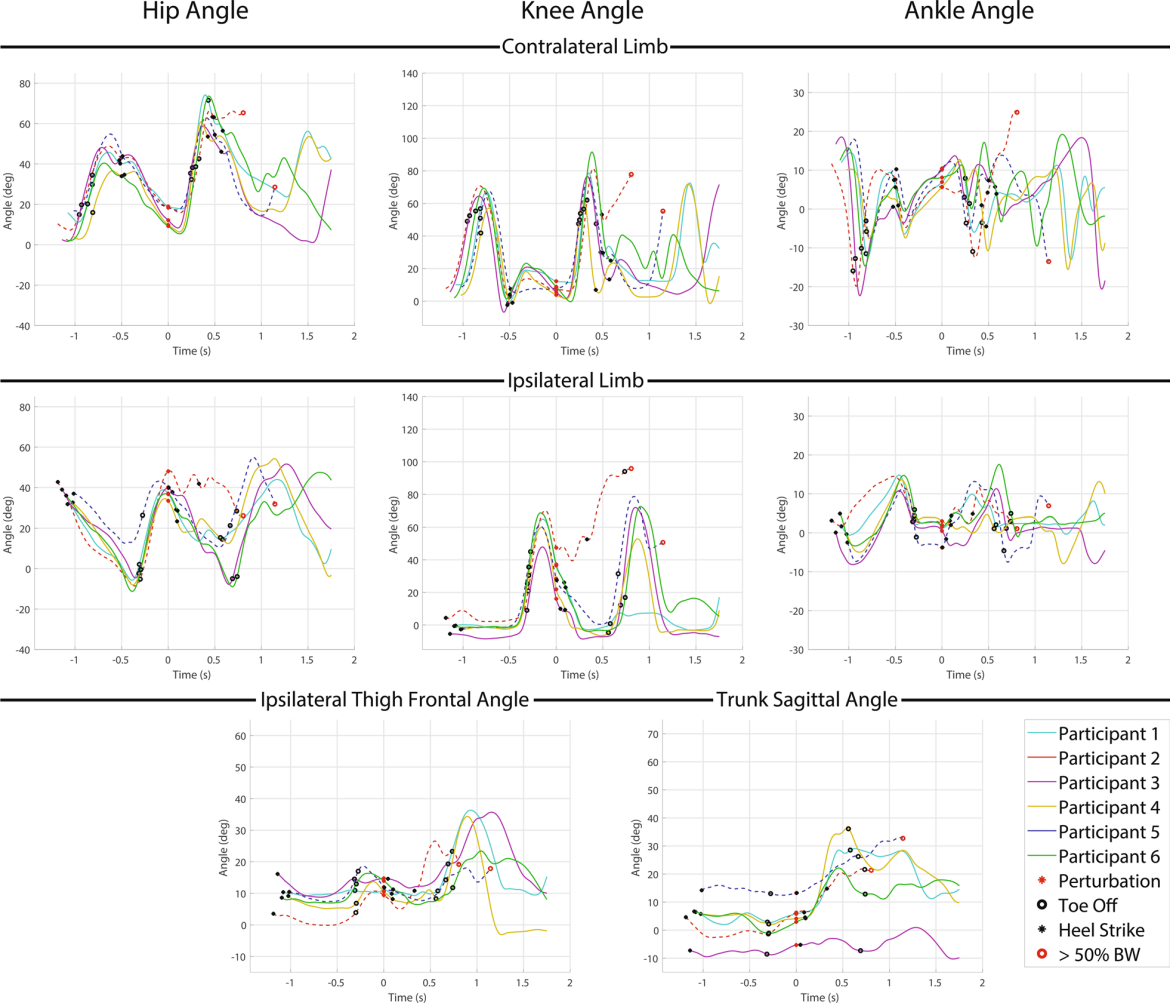
Kinematic data for late swing perturbation lowering strategy responses. Each plot depicts a different kinematic variable with the first row depicting the contralateral limb (i.e., support limb) hip, knee and ankle angle from left to right, the second row depicting ipsilateral limb (i.e., perturbed limb) hip, knee, and ankle angle from left to right, and the third row depicting ipsilateral thigh frontal plane angle (i.e., abduction) and trunk sagittal plane angle from left to right. Warm colored lines (red and yellow) depict non-MPK users and cool lines (cyan, purple, blue and green) depict C-Leg users. Solid lines represent a successful recovery, while dashed lines represent a fall. The red circle represents when the perturbation occurs, the black circles represent heel strikes, the black rings represent toe offs, and the red rings represent when a fall occurs

The lowering strategy was the only recovery strategy used by participants in late swing phase. Hopping was observed during 2 of the 6 lowering strategies (33%), 1 of which was a fall (50%). Kinematic trajectories for the lowering strategy are presented in Fig. 8. Both falls resulted from knee yielding upon loading due to inadequate extension. The non-MPK user (P2) fell due to poor stance support resulting in the knee buckling while lowering, while the C-Leg user (P5) fell during the following recovery step due to insufficient knee extension before loading. For all non-fallers, knee extension resulted in a knee configuration less than $30^{\circ }$ flexion at ground contact. Additionally, all non-fallers used thigh abduction to cross the obstacle (avg: $32.4^{\circ }$).

#### Recovery metrics

**Fig. 9:**
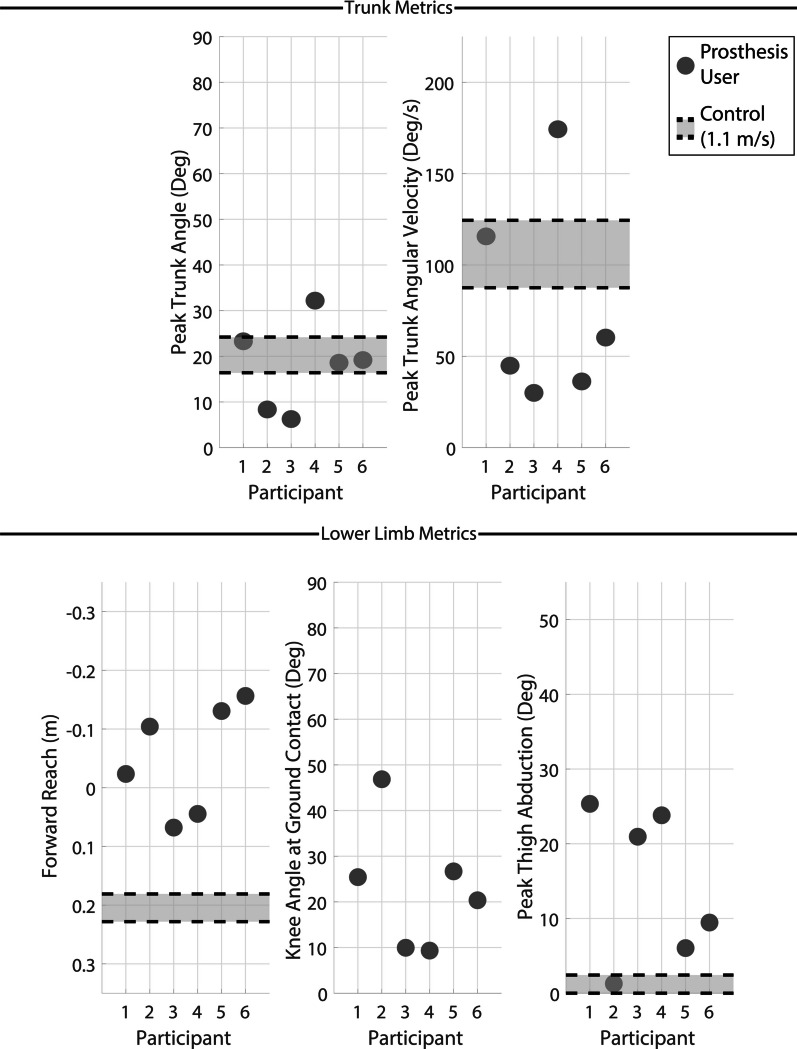
Key kinematic recovery outcome metrics for late swing perturbations. The top row displays peak trunk angle (left) and peak trunk angular velocity (right). The bottom row displays forward reach (body CoM to foot CoM, left), knee angle at first ground contact (middle) and peak thigh abduction (right). All metrics except knee angle are normalized to the value at the perturbation and are measured between the perturbation and the final recovery ground contact (first for elevating and second for delayed lowering and lowering). Knee angle is measured at first ground contact. Forward reach has an inverted vertical axis to maintain the visual trend across the plots (i.e., markers positioned lower on the plots indicate a more favorable outcome). The light grey region bordered in black dashed lines indicate the interquartile range of the control group data at 1.1 m/s from a previous study [20, 23]

Trunk recovery metrics are presented in the top row of Fig. 9 with control data for comparison. Trunk deviations following late swing perturbations were low compared to early and mid swing.

Lower limb metrics are reported in the bottom row of Fig. 9 with control data for comparison. Forward reach is low with most transfemoral prosthesis users having forward reach near 0 m or below. Knee flexion at ground contact is much lower in late swing phase than early and mid swing with only one trial above $30^{\circ }$ due to the proximity of the perturbation to the end of swing phase. However, high peak thigh abduction is still present since circumduction is still required to avoid the obstacle during recovery. Both trunk and lower limb metrics for late swing are summarized in Table 4.

**Table 4:** Late swing perturbation outcomes

Participant	Fall?	% Swing	Strategy	Trunk ang	Trunk ang vel	Forward reach	Knee ang	Thigh abd
P1	No	62%	L	$23.3^{\circ }$	$115.7^{\circ }$/s	− 0.02 m	$25.4^{\circ }$	$25.3^{\circ }$
P2	Yes	60%	L w/ hop	$8.4^{\circ }$	$44.9^{\circ }$/s	− 0.10 m	$46.9^{\circ }$	$1.3^{\circ }$
P3	No	64%	DL	$6.3^{\circ }$	$30.0^{\circ }$/s	0.07 m	$10.0^{\circ }$	$20.9^{\circ }$
P4	No	67%	DL	$32.2^{\circ }$	$174.3^{\circ }$/s	0.04 m	$9.3^{\circ }$	$23.8^{\circ }$
P5	Yes	67%	L	$18.6^{\circ }$	$36.2^{\circ }$/s	− 0.13 m	$26.7^{\circ }$	$6.0^{\circ }$
P6	No	61%	L w/ hop	$19.2^{\circ }$	$60.3^{\circ }$/s	− 0.16 m	$20.3^{\circ }$	$9.5^{\circ }$

## Discussion

### Recovery strategies and causes of falls

Based on the fall outcome results, this work builds on the limited body of prior experimental studies on transfemoral prosthesis users’ stumble recovery response and suggests they face a higher risk of falling from stumble perturbations than controls. Only a single transfemoral prosthesis user participant recovered from all (3) perturbations, while no falls were reported for seven control participants across 190 trials in total at 1.1 m/s using the same experimental methodology [20].

The assertion that the risk of falling is higher in transfemoral prosthesis users is further supported by the primary recovery metrics: peak trunk angle, peak trunk angular velocity, forward reach, and knee angle at ground contact. By comparing the metrics to the control participants’, we can highlight where the performance of the transfemoral prosthesis users has the greatest deficits. However, it is important to note the limitations of statistical analysis due to the low sample size in this preliminary case series. Trunk angle and angular velocity were significantly higher in transfemoral prosthesis users relative to controls in early swing phase (trunk angle: p < 0.001, trunk angular velocity: p < 0.001), demonstrating a large disparity in trunk control where the greatest fall rate was seen. The disparity is likely due to the mismatch in recovery strategy between the two groups (i.e., elevating versus delayed lowering) leading to a longer, delayed recovery for the transfemoral prosthesis users which requires the complete reversal of the momentum of the perturbed limb. In mid and late swing, the trunk metrics were generally similar between transfemoral prosthesis users and controls, apart from trunk angle in mid swing (p = 0.029). However, the disparity in forward reach remained high for transfemoral prosthesis users compared to the controls across all three regions of swing (p < 0.001 for all three regions), suggesting general difficulty in adjusting their base of support to recapture CoM support following the perturbations. The reduced forward reach may be due to delayed recovery step initiation from difficulty surmounting the obstacle which requires circumduction due to the lack of knee flexion, especially in early swing where the elevating strategy is generally unavailable. The loss of balance may also contribute to the delayed swing initiation. The delay in initiating the recovery step decreases the available time to adjust the base of support which may limit forward reach. Finally, the knee flexion angle at first ground contact was greatest in early swing, followed by mid swing and late swing. Therefore, the earlier the perturbation is in swing phase, the higher the risk of the knee buckling upon heel strike. With all four metrics demonstrating worse performance in early swing (peak trunk angle, peak trunk angle velocity, forward reach, and knee angle at ground contact), three metrics in mid swing (all except trunk angular velocity), and one in late swing (forward reach), the trend in the recovery metrics follows the trend seen in fall outcome.

Following early swing perturbations, knee buckling was frequently experienced (3 out of 7 falls). Additionally, an inability to complete the recovery strategy in time before losing their body CoM support (i.e., falling forward) was an issue for some participants (6 out of 7 falls). The elevating strategy led to better recovery outcomes when successful, resulting in a single stride recovery with less trunk deflection and thigh abduction compared to the delayed lowering strategy. However, as the elevating strategy was only successfully completed one time by a single non-MPK participant, it may be challenging to perform due to the required swing knee flexion and careful coordination to ensure landing after the knee is fully extended. Using the delayed lowering strategy in early swing phase was more common and required an immediate reversal of the perturbed leg’s momentum as well as an additional recovery step, and results in a longer overall recovery time with more trunk deflection and thigh abduction (i.e., hip circumduction) due to difficulty initiating the knee flexion for the next step on the prosthesis side and difficulty avoiding the obstacle. The deficient knee flexion and required circumduction can also lead to falls as it can result in a delayed recovery, further loss of balance or, in the case that the circumduction is insufficient, the foot remaining caught on the obstacle.

For mid and late swing perturbations, poor stance support was the cause of all six falls as the knee buckled on ground contact. When the lowering/delayed lowering strategies were successful, thigh abduction was still required to avoid the obstacle due to difficulties initiating swing knee flexion during the recovery step on the prosthesis side, with inadequate thigh abduction playing a role in one of the falls.

The outcomes in mid to late swing generally matched previous overground [15] and treadmill-based [24] works which observed primarily the delayed lowering or lowering strategy being used in response to mid to late swing perturbations. Additionally, the overground study found that landing with a greater knee flexion angle led to the one observed fall, which aligns with the observations of this preliminary case series. However, this case series only observed two elevating strategies during very early swing perturbations (<30%), while the treadmill-based study reported a much higher prevalence from early to mid swing phase. This may be due to differences in methodologies between the studies (i.e., the previous study used rope-blocking to emulate an obstacle and allowed the use of handrails). Unsurprisingly, the skipping sub-strategy, which was only observed in the previous treadmill-based study during sound limb perturbations, was not observed in this case series. Interestingly, the hopping sub-strategy was more prevalent in this case series than either previous study. The overground study only reported hopping during sound limb perturbations. The treadmill study reported a few instances of hopping throughout swing during sound limb perturbations or in early swing during prosthetic limb perturbations. However, in the current study, hopping was observed frequently throughout swing phase, but predominately during early swing and most often associated with a fall. Hopping was only used during delayed lowering or lowering strategies as a means to clear the obstacle or adjust the participant’s base of support without relying on the prosthetic limb, suggesting it may act as a compensation strategy due to inadequate functionality in the prosthetic limb.

### Proposed interventions

In these trials, falls appeared to be related to the following functional deficits in the various knee prostheses: (1) insufficient resistance to stance knee flexion upon ground contact (i.e., buckling due to insufficient stance support); (2) insufficient swing extension ensuring the knee is adequately extended when landing after a perturbation; (3) difficulty initiating swing flexion following a perturbation, forcing a reliance on hip circumduction; and (4) paradoxically, excessive swing flexion impedance in early swing, which prevents the potential utilization of the elevating strategy. These four elements describe the deficits of the prostheses across the entire cohort; however, the extent of each deficit varied with prosthesis type.

While this preliminary case series was not constructed to directly compare the functionality of MPKs relative to non-MPKs, as such a study would employ both devices on the same set of participants, some interesting initial trends have emerged with regard to prosthesis type which generally align with previous reports of the inferior performance of non-MPKs during stumbles [11, 12, 14, 19]. While the low sample size and heterogeneity of the participants in this study limit the ability to make comprehensive comparative observations on the efficacy of MPKs versus non-MPKs, these initial trends demonstrate an interesting dichotomy in performance between the two types of prosthetic devices. Therefore, any observations described herein should be considered with the significant caveat that the response of the device may not be entirely isolated from the response of the participant wearing it.

With that caveat noted, it was observed that insufficient stance support (Deficiency 1) affected both MPK and non-MPK users, but appeared to be a more substantial problem for non-MPK users, who often had their knees buckle underneath them during landing. One MPK user experienced knee buckling when landing in a flexed position during all 5 falls; however, across both non-MPK users, 4 out of 6 falls involved the knee buckling at some point during the recovery.

Inadequate swing extension assistance (Deficiency 2) is related to insufficient stance support, as increased swing extension assistance could help ensure the knee is sufficiently extended before loading, thereby reducing the flexion moment arm about the knee to avoid buckling. Non-MPK users utilized hip motion during swing to ensure adequate knee extension during elevating strategies, and both non-MPK and MPK users often continued extending their hips after ground contact during lowering strategies. Based on the rate of knee buckling, continuing or maintaining knee extension after ground contact proved more difficult for the non-MPK users; however, swing extension shortcomings were common in both classes of prosthesis, even if it did not directly lead to falls for most MPK users.

Poor initiation of swing flexion (Deficiency 3) was equally an issue for both device classes, as users of both utilized thigh abduction during the prosthesis-side recovery stride when using the delayed lowering or lowering strategies. Thigh abduction was required as there was otherwise insufficient toe clearance to overcome the obstacle from knee flexion alone. The inadequate swing knee flexion was due to the difficulty of initiating swing while off balance. Falls due to this difficult next step (6 out of 13) were either the result of poor balance and slow recovery step time while attempting circumduction around the obstacle, or a complete lack of circumduction causing the foot to remain caught on the obstacle.

Finally, excessive impedance against swing flexion (Deficiency 4) paradoxically can interfere with the ability to recover from a stumble, specifically in early swing phase by preventing the use of the elevating strategy. A lack of flexion was exclusively observed in the MPK user group as they were the only group which was unable to perform an elevating strategy, presumably due to the C-Leg stumble recovery behavior, which offers an increased resistance to knee flexion following the perturbation. While such flexion resistance is important in stance for successfully performing the lowering strategy, using the lowering strategy in response to an early swing perturbation often resulted in a fall (6 out of 9 lowering attempts).

Various functional improvements that address these four deficits could potentially mitigate the effect of stumble perturbations and potentially prevent falls. First, robust stance support is required to ensure the knee does not buckle as recoveries often require the user to land with some amount of knee flexion or behind their CoM. Many prostheses provide stance-knee stability at heel strike that must also accommodate stance-knee yielding for stair and slope descent and stand-to-sit transitions. If knee prostheses were to implement a stumble-specific amount of stance-knee support during the step following a perturbation, the increased knee support could potentially aid in stumble recovery. Increased stance support could be implemented through passive (e.g., hydraulic resistance) or powered (e.g., electric motors) means. While the passive implementation of stumble-specific stance-knee support may be feasible in MPKs, non-MPKs may still benefit from an increased baseline stance support as long as it does not impede other functionality.

Improved swing extension assistance may also aid recovery by preventing knee buckling. Improved extension can be achieved without the need for perturbation detection by using increased or variable stiffness extension springs in passive prostheses, or through powered means by assisting the swing trajectory via an electric motor. An improved extension aid might reduce the need for increased stance-knee support in non-MPKs, although this will reduce the magnitude of swing flexion.

Assisting swing knee flexion initiation following the perturbation despite the presence of a stumble object in front of the toe may also improve likelihood of recovery. Increased knee flexion would reduce the need for thigh abduction due to the inability to initiate a ballistic swing. Improved swing initiation is difficult to solve in passive systems as the issue comes from the user’s inability to move through the range of motion required to initiate the prosthesis’s ballistic trajectory. However, powered solutions may enable improvement by providing knee flexion without the prerequisite motion of the thigh that passive prostheses require.

Finally, reduced swing flexion impedance following a perturbation in early swing phase could allow sufficient deflection of the prosthesis after contact with the obstacle to perform an elevating strategy. However, a robust extension aid in either a passive or powered system would also be required to protect from excessive flexion resulting in the knee buckling upon landing. A stumble-specific reduction in swing flexion resistance for MPKs may be an effective way to implement this behavior in passive devices. Closed-loop trajectory control or tuned, feedforward knee extension torque in a powered device could also potentially provide the elevating strategy behavior without jeopardizing stance support.

Beyond device-based interventions, strength [32] and balance and perturbation [16, 33] training interventions may be beneficial. Strength training for the core and the sound limb hip may improve trunk and CoM control and the stability of the individual while they manipulate their prosthetic limb in swing following a perturbation. Additionally, strengthening sound limb ankle plantarflexion may enable increased CoM height and prosthetic toe clearance to aid in obstacle crossing while limiting hip hiking and circumduction. Strengthening the prosthetic limb hip joint may also improve the ability to complete knee extension motions to limit the likelihood of knee yielding upon ground contact (Deficiency 1 and 2) and to initiate swing flexion following a perturbation (Deficiency 3).

Balance and perturbation training may be able to improve user coordination with their device through exposure to and practice in stumble recovery scenarios. Specific coordination skills may include ensuring the knee is driven to extension during swing or immediately following ground contact (Deficiency 1 and 2), initiating swing phase following a perturbation (Deficiency 3), and using an adequate amount of circumduction to avoid an obstacle. Training may be a particularly robust method for improving swing initiation following a perturbation for passive prosthesis users as passive design modifications are not well equipped to assist with this deficiency. Future work could look into the effects of training on transfemoral prosthesis users’ ability to recover from stumble perturbations, as any analysis of this effect is beyond the scope of this work due to the limited number of participants and perturbations.

### Limitations

Several limitations of this preliminary case series remain to be addressed. First, while this data set is among the most comprehensive of laboratory studies to date, more data will further improve the understanding of stumble recovery behavior across a very heterogeneous population with heterogeneous knee prostheses.

Additionally, the perturbation system in this work utilizes a treadmill-based obstacle delivery apparatus, which enables a large number of realistic, precisely-timed perturbations. Unlike overground perturbations, however, participants are unable to stop during or immediately after their recovery in an attempt to regain their balance due to the constant velocity of the treadmill. While the constant velocity provides an inertial reference frame without dramatically altering the stumble recovery response in controls [20], it also imposes a loose locational constraint on the participant. While this constraint is not unreasonable, since continuing to walk following a perturbation is a common response in controls, further study is needed to determine whether it might affect recovery outcomes in transfemoral prosthesis users. Additionally, the same treadmill velocity was used for all participants to keep inter-participant comparisons consistent; however, this also may have impacted the recovery response since it may not have matched each participant’s preferred walking speed. Also, the use of the overhead harness may have had an effect on participants’ stumble recovery response, particularly if they felt they could rely on the harness to prevent a fall.

The use of Serial Sevens as a distraction task may have impacted the stumble recovery response. While the distraction task was methodologically necessary to avoid unnatural compensatory movements during steady-state walking as observed in pilot testing, it may have led to variations in the stumble recovery response that otherwise may not be present in real-world stumble recovery. However, a recent publication found that Serial Sevens did not notably alter the first step of the recovery response during balance and walking perturbations in non-prosthesis users, potentially due to the reflexive nature of the response [34]. Also, studies characterizing the circumstances of real-world stumbles have noted that stumbles often occur while distracted, so the addition of a secondary task such as Serial Sevens may be akin to real-world perturbations [35, 36].

Lastly, the comparison to the control group at 1.1 m/s rather than 0.8 m/s may have resulted in differences in the stumble recovery response between the control group and the transfemoral prosthesis user group due to a difference in stride period which alters the time available to adjust the base of support. However, preliminary results in controls have found similar stumble recovery responses across 0.8 m/s and 1.4 m/s [23].

## Conclusions

This paper presents a study intended to better understand the nature of stumble recovery for transfemoral prosthesis users. The study involved six transfemoral prosthesis users, who were stumbled on the prosthesis side in aggregate 24 times across early, mid, and late swing phase. Results indicate that transfemoral prosthesis users are in general much more susceptible to stumble perturbations than controls, particularly for early and mid-swing perturbations. Specifically, five of six transfemoral prosthesis users experienced a fall (defined as greater than 50% body weight in the overhead harness), with two participants falling every time. Across all participants, 13 of the 24 (54%) stumble perturbations resulted in a fall; 7 out of 11 (64%) were in early swing; 4 out of 7 (57%) were in mid swing; and 2 out of 6 (33%) were in late swing. Using the same apparatus and protocol (albeit at higher walking speed), seven control participants collectively exposed to 190 perturbations experienced no falls. Four key metrics (peak trunk angle, peak trunk angular velocity, forward reach, and knee angle at ground contact) used to assess quality of recovery reinforced this trend, as early swing perturbations resulted in the greatest number of deficient metrics compared to controls (all four) followed by mid swing with three (all except trunk angular velocity) and then late swing with one (forward reach), mirroring the trend in falls. Based on experimental measurement and observation of the trials resulting in falls, the authors identify four potential prosthesis deficits leading to falls: (1) insufficient resistance to stance knee flexion; (2) insufficient swing extension; (3) difficulty initiating swing flexion following a perturbation; and (4) excessive swing flexion impedance in early swing preventing the utilization of the elevating strategy. Addressing these deficiencies through revisions to prosthetic design and tuning or the implementation of strength or perturbation and balance training could potentially improve quality of recovery and reduce the likelihood that a stumble perturbation will result in a fall for transfemoral prosthesis users.

### Supplementary Information

Additional file1. Stumble Recovery Response Video.

## Data Availability

Data from the study are available from the corresponding author on reasonable request.

## Abbreviations

MPK: Microprocessor knee
GRF: Ground reaction force
CoM: Center of mass
P1-P6: Participant 1–Participant 6
E: Elevating
DL: Delayed lowering
L: Lowering
